# CircXRN2 accelerates colorectal cancer progression through regulating miR-149-5p/MACC1 axis and EMT

**DOI:** 10.1038/s41598-024-52257-3

**Published:** 2024-01-30

**Authors:** Pan-Feng Feng, Long-Xun Zhu, Nan Sheng, Xin-Shuai Li, Pei-Gen Liu, Xiang-Fan Chen

**Affiliations:** 1 Department of Pharmacy, Nantong First People’s Hospital and Affiliated Hospital 2 of Nantong University, Nantong, China; 2 Institute of Experimental and Clinical Immunology, Nantong First People’s Hospital and Affiliated Hospital 2 of Nantong University, Nantong, China; 3 Department of General Surgery, Central Hospital of Panzhihua City, Panzhihua, 617000 Sichuan China; 4 Biological Sample Bank, Nantong First People’s Hospital and Affiliated Hospital 2 of Nantong University, No. 666, Shengli Road, Nantong, 226001 Jiangsu China

**Keywords:** Cancer, Cell biology

## Abstract

In China, there has been a persistent upward trend in the incidence and mortality rates of colorectal cancer (CRC), with CRC ranking second in incidence and fifth in mortality among all malignant tumors. Although circular RNAs (circRNAs) have been implicated in the progression of various cancers, their specific role in CRC progression remains largely unexplored. The objective of this study was to elucidate the role and underlying mechanisms of circXRN2 in CRC. Differential expression of circXRN2 was identified through whole transcriptome sequencing. The expression levels of circXRN2 and miR-149-5p were quantified in CRC tissues, corresponding adjacent normal tissues, and CRC cell lines using quantitative reverse transcription-polymerase chain reaction (qRT-PCR). The stability of circXRN2 was confirmed through RNase R and actinomycin D experiments. The binding interaction between circXRN2 and miR-149-5p was validated through RNA pull-down, RNA immunoprecipitation, and dual-luciferase assays. The biological functions of circXRN2 were assessed through a battery of in vitro experiments, including the CCK-8 assay, EdU assay, scratch assay, Transwell assay, and flow cytometry assay. Additionally, in vivo experiments involving a tumor transplantation model and a liver-lung metastasis model were conducted. The influence of circXRN2 on the expression of epithelial–mesenchymal transition (EMT)-related genes was determined via Western blotting analysis. In CRC tissues and cells, there was an upregulation in the expression levels of both circXRN2 and ENC1, while miR-149-5p exhibited a downregulation in its expression. The overexpression of circXRN2 was found to enhance tumor proliferation and metastasis, as evidenced by results from both in vitro and in vivo experiments. Functionally, circXRN2 exerted its antitumor effect by suppressing cell proliferation, migration, and invasion while also promoting apoptosis. Mechanistically, the dysregulated expression of circXRN2 had an impact on the expression of proteins within the EMT signaling pathway. Our results demonstrated that circXRN2 promoted the proliferation and metastasis of CRC cells through the miR-149-5p/ENC1/EMT axis, suggesting that circXRN2 might serve as a potential therapeutic target and novel biomarker in the progression of CRC.

## Introduction

Colorectal cancer (CRC) stands as the second most lethal cancer globally and ranks as the third most prevalent malignancy^1,2^. In China, both the incidence and mortality rates of CRC continue to follow an upward trend. As per the 2020 China Cancer Statistics Report, CRC occupies the second position in terms of incidence and the fifth position in mortality rates among all malignant tumors. Specifically, there were 555,000 new cases and 286,000 CRC-related deaths in 2020^2^. Given its high incidence, unfavorable prognosis, and the common late-stage diagnosis of the majority of patients^3–5^, it is of utmost importance to delve into and gain a profound understanding of the mechanisms propelling CRC progression.

Furthermore, the identification of molecular drivers of CRC progression not only facilitates more accurate prognostic assessments but also holds the potential for more efficient targeted therapies, thereby enhancing clinical management. Consequently, the quest for early diagnostic markers and the exploration of key molecular players involved in CRC growth and metastasis constitute current research focal points.

Circular RNAs (circRNAs) constitute a novel category of non-coding RNAs characterized by their distinctive covalently closed, continuous loop structure, which lacks the 5′ cap and 3′ polyadenylated tail^6^. Dysregulation of circRNAs has been closely linked to cancer progression, particularly in the context of CRC. Studies have revealed that circRNAs harbor multiple miRNA binding sites, functioning as endogenous miRNA sponges, a concept referred to as competitive endogenous RNAs (ceRNAs). By modulating the interactions between miRNAs and mRNAs, circRNAs have been implicated in diverse processes, including proliferation, apoptosis, migration, and invasion in CRC^7–9^. Despite the increasing recognition of functional circRNAs in various cancer types through high-throughput sequencing and bioinformatics approaches, their mechanisms remain incompletely understood^10^. Notably, limited research has been dedicated to investigate the functional roles and mechanisms of circRNAs involved in CRC metastasis^11^.

Through whole transcriptome sequencing of five groups of CRC tissues and corresponding adjacent normal tissues, we observed a significant upregulation of circXRN2 expression in CRC tissues compared to adjacent normal tissues. However, the specific role of circXRN2 in the progression of CRC remains unclear.

In this study, we unveiled the regulatory mechanism of circXRN2 in CRC. The expression of circXRN2 was markedly increased in CRC tissues and cells, and both in vitro and in vivo experiments confirmed its role in promoting CRC proliferation and metastasis. Importantly, we identified that circXRN2 served as a sponge for miR-149-5p, thereby post-transcriptionally regulating the expression of ENC1. Furthermore, our investigations revealed that depletion of circXRN2 could inhibit key proteins involved in the epithelial–mesenchymal transition (EMT) process. In summary, our findings collectively indicated that circXRN2 suppressed CRC progression through the miR-149-5p/ENC1/EMT axis. circXRN2 might hold promise as a potential biomarker and therapeutic target in CRC.

## Materials and methods

### Clinical specimen collection

All methods were performed in accordance with the relevant guidelines and regulations. A total of 100 paired samples, consisting of CRC tissues and adjacent normal tissues, were obtained from the First People's Hospital of Nantong and the Central Hospital of Panzhihua, spanning from December 2019 to June 2023. These patients did not receive chemotherapy or radiotherapy prior to surgery. Ethical approval for this study was obtained from the Clinical Research Ethics Committee of the First People's Hospital of Nantong, and informed consent was obtained from each patient prior to their participation.

### Cell culture

CRC cell lines, namely HCT116, SW620, and DLD-1, along with normal colon epithelial cells (NCM460), were obtained from the Cell Bank of the Chinese Academy of Sciences. SW620 cells were cultured in L-15 medium, DLD-1 cells in RPMI-1640 medium, and both NCM460 and HCT116 cells in DMEM. All culture media were supplemented with 10% fetal bovine serum and 1% penicillin–streptomycin solution, and the cells were maintained at 37 °C in a humidified atmosphere containing 5% CO_2_.

### RNA extraction and quantitative real-time PCR (qRT-PCR)

Total RNA from tissues and cells was extracted using the TRIzol Reagent kit following the manufacturer’s instructions, and the quality and concentration of RNA were assessed using NanoDrop 2000. qRT-PCR was performed using ABI SYBR Green Master Mix (Invitrogen). 18S rRNA and U6 were used as internal controls, and the relative expression levels of XRN2, circXRN2, miR-149-5p, and ENC1 were calculated using the 2^−ΔΔCT^ method. Primer sequences are provided in Table 1.

**Table 1:** Primer sequences of XRN2, circXRN2, miR-149-5p, ENC1 and GADPH.

Name	F	R
XRN2	ACCAGCTTTCACTCCTAG	TTCCCATAACCTGACATT
circXRN2	AAGCAGCCTATGAAATGA	ACTTGAGAACCTGGTGAA
miR-149-5p	ACAGGCACCGAGACAACACACCC	GCTGTCAACGATACGCTACGTAACG
ENC1	GCAGTCGCTACTTTGAGG	TGGGTGGATGGAATTGTC
GADPH	GGAGCGAGATCCCTCCAAAAT	GGCTGTTGTCATACTTCTCATGG

### Transient transfection of cells

CRC cell lines were seeded into 6-well plates at a density of 1 × 10^5^ cells per well and cultured under standard conditions in a cell culture incubator at 37 °C until they reached 60% confluence. Following the guidelines outlined in the Lipofectamine 2000 manual, serum-free medium containing the appropriate plasmids and vectors was introduced into the 6-well plates. Transfected cells were harvested at 24, 48, or 72 h post-transfection, and the effectiveness of plasmid expression was evaluated through qRT-PCR for subsequent analyses.

### Dual-luciferase assay

The binding sites of circXRN2 with miR-149-5p were predicted utilizing the Starbase database. Wild-type and mutant DNA fragments of circXRN2 were generated and then inserted into the pMIR-GLo luciferase reporter vector. Subsequently, SW620 and HCT116 cells were co-transfected with either the miR-149-5p mimic or miR-149-5p NC sequences, in addition to the wild-type or mutant circXRN2 gene fragments, using Lipofectamine 2000, following the manufacturer's instructions. After a 48-h transfection period, luciferase activity was quantified using the Dual-Glo^®^ Dual-Luciferase Reporter Gene Assay System (Promega (e2920)). Relative luciferase activity was determined as the ratio of firefly luciferase activity to Renilla luciferase activity.

### RNA pull-down assay

To explore the association between circXRN2 and miR-139-5p as well as miR-149-5p, an RNA pull-down assay was carried out. Biotin-labeled circXRN2 probes were incubated with streptavidin magnetic beads at room temperature for 2 h, resulting in the generation of probe-coated beads. SW620 and HCT116 cells were lysed using RIPA lysis buffer. The lysates were subsequently subjected to incubation with the probe-coated beads at 4 °C overnight, followed by a series of washing steps and subsequent RT-qPCR experiments.

### RNA immunoprecipitation (RIP) assay

RIP analysis was conducted employing the Magna RIPTM RNA-Binding Protein Immunoprecipitation Kit. HCT116 and SW620 cells were transfected either with the miR-149-5p mimic or negative control. After a 48-h incubation, the cells were lysed using a complete RNA lysis buffer. Subsequently, magnetic beads conjugated with a human anti-Argonaute2 (Ago2) antibody or a negative control IgG were introduced into the cell lysates, along with RIP buffer. The lysates were then subjected to overnight rotation. On the following day, after a 30-min incubation with proteinase K, RNA extracted from the immunoprecipitation process was obtained. Finally, the enrichment of circXRN2 was evaluated using qRT-PCR.

### Actinomycin D experiment

HCT116 and SW620 cells were seeded in 6-well plates and treated with actinomycin D to achieve a final concentration of 3 μg/mL. After incubating in a cell culture incubator for 0, 4, 8, 12, and 24 h, total RNA was extracted from the cells, and the expression levels of circXRN2 and XRN2 mRNA were assessed by RT-qPCR.

### RNase R experiment

RNA was extracted from HCT116 and SW620 cell lines, and RNase R was added for RNA digestion. The reaction was prepared with RNase R at a concentration of 3 U/μg RNA in a 20-μL reaction system. After incubation at 37 °C for 30 min, circXRN2 and linear XRN2 mRNA expression were analyzed by qRT-PCR.

### Nuclear-cytoplasmic fractionation

Nuclear and cytoplasmic RNA were isolated following the guidelines outlined in the PARISTM kit. Upon cell lysis, centrifugation was conducted, resulting in the separation of the supernatant containing cytoplasmic RNA and the pellet containing nuclear RNA. The extracted RNA was subsequently subjected to purification and cleaning using a filtration column. Following this, RT-qPCR was performed to ascertain the presence of circXRN2 in either the cytoplasm or the nucleus.

### Construction and evaluation of subcutaneous tumor models and metastatic mouse models

The study is reported in accordance with ARRIVE guidelines. All animal experiments were approved by the Ethics Committee for Animal Experiments of Nantong university. Subcutaneous tumor model: Four-week-old SPF female BALB/c-nu nude mice were carefully chosen and given 1 week for acclimatization. These mice were then divided into two groups: a control group and a circXRN2 knockdown group, with five mice allocated to each group. Cells transduced with the respective groups were expanded, cultured, and subsequently resuspended to achieve a concentration of 1 × 10^8^ cells/mL. Each mouse was subcutaneously injected with 1 × 10^6^ cells (100 µL) into the dorsal area. Following this, tumor growth was meticulously monitored, and tumor volume was measured every 7 days, spanning a total duration of 35 days. At the conclusion of the experiment, the animals, which received an intraperitoneal injection of ketamine (100 mg/kg) prepared with a physiological saline solution by cervical dislocation were humanely euthanized. Photographs of the tumors were captured, and both tumor volume and weight were recorded. Tumor volume was computed using the formula V = (length × width^2^)/2.

#### Metastatic mouse model

Nude mice were employed for the tail vein injection of HCT116 cells, with each group receiving 1 × 10^6^ cells and consisting of five mice. Subsequently, after a 60-day incubation period, the mice were euthanized. Photographs of the lungs and liver were taken to document the count of metastatic nodules in these organs. The identification of hepatic and pulmonary metastatic nodules was achieved through the use of the H&E staining method. Microscopic examination was performed, and the results were reported as an average of three counts. Ki67 expression was assessed through immunohistochemistry.

### CCK-8 assay

The proliferation of tumor cells was assessed utilizing the CCK-8 assay. Cells prepared according to the experimental groups underwent digestion with 0.25% trypsin, followed by centrifugation. Subsequently, they were seeded at a density of 5 × 10^3^ cells per well in a 96-well plate. These cells were cultured in a 37 °C incubator with a 5% CO_2_ atmosphere. Each group was established with six replicates. After the specified experimental duration, 10 μL of CCK-8 culture solution was introduced into each well. Following a 2-h incubation in the cell culture incubator, the optical density of each well was measured at 450 nm using an enzyme-linked immunosorbent assay reader, and a CCK-8 curve was generated.

### EdU assay

The EdU assay was carried out using HCT116, DLD-1, and SW620 cells post-transfection. These cells were seeded in 96-well plates and cultured until they reached the logarithmic growth phase. Subsequently, 100 μL of EdU (50 μM) culture medium was added to each well, and the cells were incubated in a cell culture incubator for 2 h. After this incubation period, the cells were washed 1–2 times with PBS for 5 min each. The cells were then fixed using polyformaldehyde at room temperature for 30 min. Following fixation, 50 μL of 2 mg/mL glycine was added, and the mixture was incubated for 5 min on a shaker. Subsequently, a 100 μL PBS wash was performed for 5 min. Next, 100 μL of PBS containing 0.5% TritonX-100 was added to each well, and decolorization was carried out on a shaker for 10 min. This step was followed by another PBS wash, lasting 5 min. Afterward, 100 μL of 1 × Apollo staining reaction solution was added to each well, and the plates were incubated in the dark at room temperature for 30 min. Following this incubation, the cells were washed 2–3 times with 100 μL PBS containing 0.5% TritonX-100, each time for 10 min. Finally, 100 μL of 1 × Hoechst 33342 reaction solution was added to each well, and the plates were once again incubated in the dark at room temperature for 30 min. Upon the completion of staining, the cells were photographed and counted under a fluorescence microscope for further analysis.

### Scratch assay

Cell migration ability was evaluated using the scratch assay. Cells from each experimental group, at the logarithmic growth phase, were seeded into 6-well plates with a cell density adjusted to 5 × 10^3^ cells/mL. When cell confluence reached approximately 90%, a 10-μL sterile tip was employed to create a uniform scratch on the plate. The cells that detached due to the scratch were subsequently washed away with PBS, and a fresh serum-free culture medium was added. Images were captured under an inverted microscope at both the 0-h and 24-h time points to monitor cell migration. The cell migration distance was calculated using image analysis, specifically: Cell migration distance = Initial scratch width − Final scratch width.

### Transwell migration and invasion assay

The Transwell assay was employed to assess cell migration and invasion capabilities, with the distinction being the presence of a matrix gel. Cells from the respective experimental groups were placed in the 24-well Transwell chambers. In the upper chamber, serum-free culture medium was added, while the lower chamber contained culture medium with 10% fetal bovine serum. After 36 h of incubation, the upper chamber cells were carefully removed using a cotton swab, followed by a PBS wash. Next, the cells were fixed with 4% polyformaldehyde for a duration of 2 h and subsequently stained with 0.1% crystal violet for 2 h. Following another wash with PBS, the chambers were inverted and allowed to air dry. Observation and imaging were conducted under an optical microscope, with four random high-power fields chosen for cell counting in order to calculate the average. This experiment was repeated three times for accuracy and consistency.

### Flow cytometry

To evaluate tumor cell apoptosis, a flow cytometry apoptosis assay was employed. CRC cells from each experimental group were resuspended in PBS to achieve a concentration of 1 × 10^6^ cells/mL. A staining working solution was prepared by adding 5 μL of FITC and 5 μL of propidium iodide (PI). In the absence of light, 1 mL of cell suspension was incubated with the staining working solution for a duration of 15 min. Apoptosis analysis was carried out within 1 h using a flow cytometer, and the apoptosis rate was subsequently calculated.

### Western blotting analysis

Cells were lysed using RIPA lysis buffer to extract total proteins, and the concentration of total protein was determined using the BCA method. A protein sample containing 100 μg was subjected to SDS-PAGE, and subsequently, the proteins were transferred to a nitrocellulose (NC) membrane via a wet transfer method. The membrane was then blocked with 5% skim milk at 4 °C for 1 h. Following the blocking step, the NC membrane was incubated with primary antibodies, including ENC1, N-cadherin, E-cadherin, vimentin, and snail, for a duration of 24 h at 4 °C. After the incubation, the NC membrane was washed three times with PBST (PBS containing Tween-20), with each wash lasting 15 min. Subsequently, the membrane was incubated with the corresponding secondary antibodies in the dark for 1 h, followed by another three PBST washes, each for 15 min. The NC membrane was scanned using an Odyssey scanner, and Image J software was employed to analyze the grayscale values of each band. The result of gels images was cropped and full-length gels and blots are included in the Supplementary Data.

### Statistical analysis

Data analysis was conducted using GraphPad Prism 9.0 data processing software. For image processing and the presentation of statistical results, Photoshop software was employed. All data were expressed as mean ± standard deviation, and statistical significance was defined as p < 0.05.

## Results

### The role of circXRN2 in CRC

**Figure 1:**
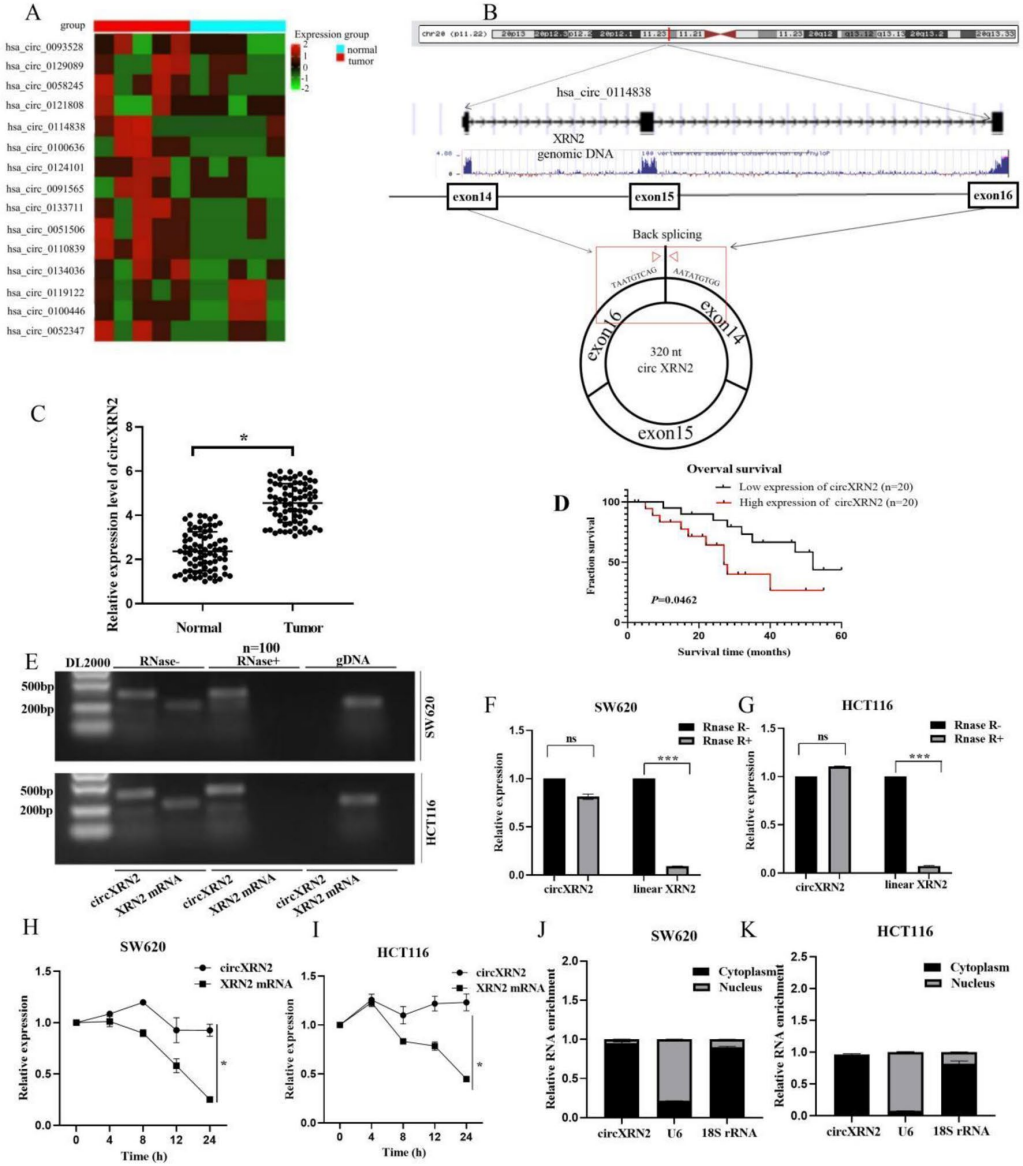
The role of circXRN2 in CRC. (**A**) Heat map of top 15 upregulated circRNAs in CRC tissues and adjacent normal tissues through whole transcriptome sequencing. (**B**) CircXRN2 forms a circular structure through exons 14, exons 15 and exons 16 in XRN2 gene. (**C**) Relative expression of circXRN2 in CRC tissues (n = 100) and adjacent normal tissues (n = 100) was detected by RT-qPCR. (**D**) Kaplan–Meier survival analysis with a Log rank test was performed to compare the overall survival rate of CRC patients with higher or lower level than median circXRN2 expression level. (**E**–**G**) Relative RNA level of circXRN2 and linear XRN2 treated with RNase R, ***p < 0.001. (**H**,**I**) Relative RNA level of circXRN2 and linear XRN2 in different time point treated with actinomycin D, *p < 0.05. (**J**,**K**) Nuclear and cytoplasmic fractions were isolated. CircXRN2 was mainly localized in cytoplasm. Experiments were performed in triplicate, and data were presented as means ± SD.

We conducted whole transcriptome sequencing on five pairs of CRC tissues and their corresponding adjacent normal tissues to identify differentially expressed circRNAs. Figure 1A illustrates the top 15 upregulated circRNAs. Among these, circXRN2 (hsa_circ_0114838) was singled out for further investigation. CircXRN2 (chr20:21319657–21324846) is derived from the XRN2 gene and results from head-to-tail splicing of the 14th-16th exons, spanning 320 base pairs, as documented in circBase (Fig. 1B). We devised a divergent primer for the specific amplification of the post-splicing form of XRN2. Subsequently, we validated circXRN2 expression in a cohort of 100 pairs of CRC tissues and corresponding non-tumor tissues using qRT-PCR. Our analysis revealed a significant increase in circXRN2 expression in CRC tissues compared to adjacent normal tissues (Fig. 1C). Moreover, circXRN2 overexpression was linked to a poorer prognosis in patients (Fig. 1D). Furthermore, we conducted a correlation analysis between circXRN2 expression and clinical characteristics in the 100 CRC patients. Chi-square test results indicated a correlation between circXRN2 expression and tumor size (p = 0.016), T stage (p = 0.001), lymph node metastasis (p = 0.012), and distant metastasis (p = 0.014) (Table 2).

**Table 2:** Correlation between clinicopathological features of colorectal cancer and expression of circXRN2.

Characteristics	Case	circXRN2 expression		*p* value
		Low	High	
Age (years)				0.130
< 60	55	21	34	
≥ 60	45	24	21	
Gender				0.492
Female	37	15	22	
Male	63	30	33	
Tumor size (cm)				0.016
≥ 5	58	34	24	
< 5	42	15	27	
T grade				0.001
T1 + T2 + T3	68	38	30	
T4	32	7	25	
Lymph node metastasis				0.012
Absent (N0)	44	26	18	
Present (N1 + N2 + N3)	56	19	37	
Distant metastasis				0.004
Absent (M0)	75	40	35	
Present (M1)	25	5	20	
Location				0.412
Colon	60	25	35	
Rectum	40	20	20	
All cases	100	45	55	

In SW620 and HCT116 cells, circXRN2 could only be amplified in cDNA but not in gDNA, eliminating the possibility of genomic rearrangement artifacts (Fig. 1E). Additionally, RNase R and actinomycin D experiments were conducted to confirm the stability of XRN2. Following RNase R and actinomycin D treatment, XRN2 mRNA levels were significantly downregulated, while circXRN2 levels remained unchanged (Fig. 1F–I). Furthermore, qRT-PCR experiments after nuclear-cytoplasmic separation confirmed that circXRN2 primarily localized to the cytoplasm (Fig. 1J–M).

### circXRN2 promotes proliferation, migration, and invasion and inhibits apoptosis in CRC cells in vitro

**Figure 2:**
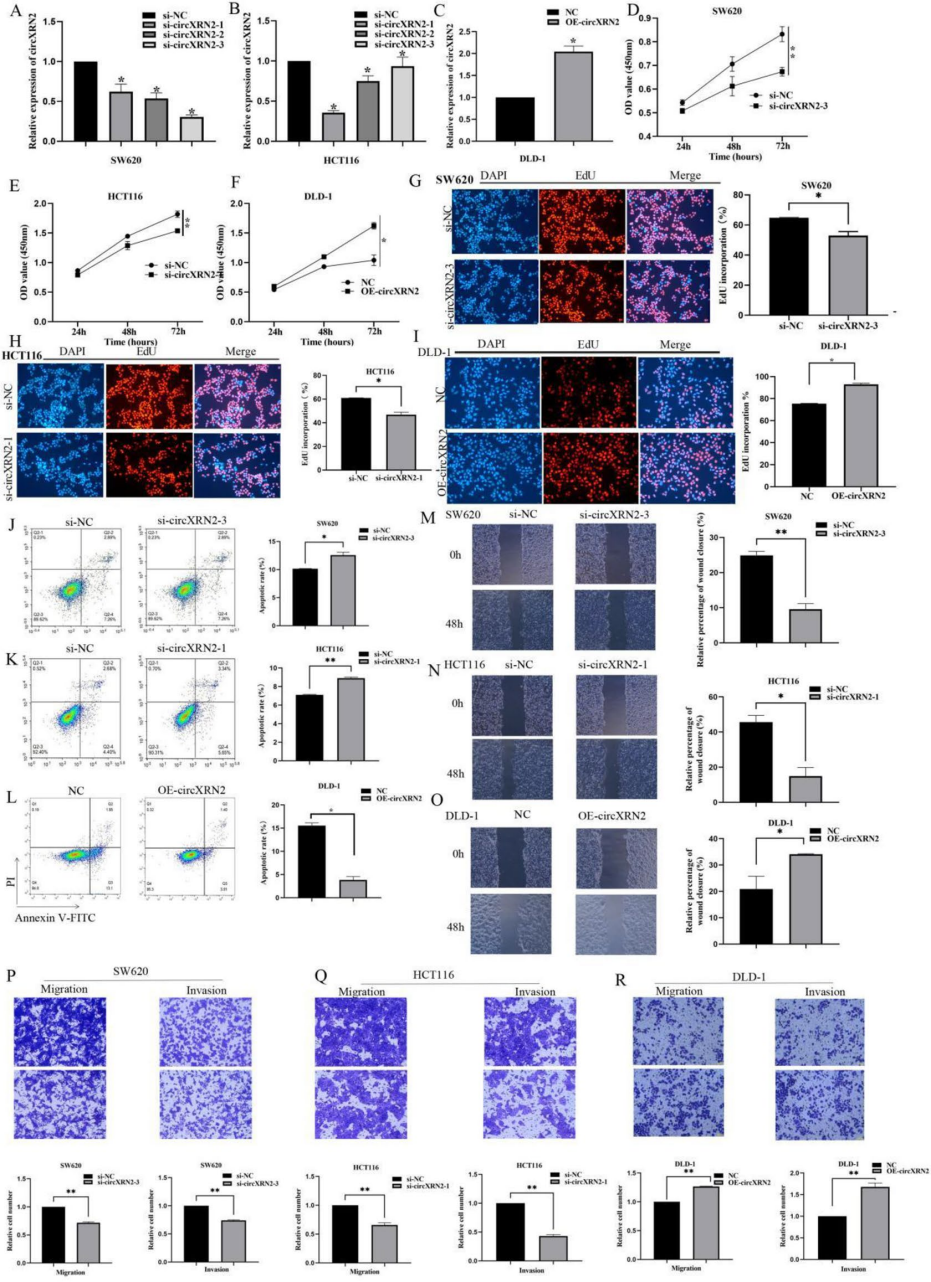
circXRN2 promotes proliferation, migration, and invasion and inhibits apoptosis in CRC cells in vitro. (**A***–***C**) qRT-PCR analysis of circXRN2 expression in SW620, HCT116 and DLD-1 cells transfected with circXRN2 siRNAs and circXRN2 plasmid. (**D***–***F**) CCK8 assays showed that knockdown of circXRN2 induced the repression of viability of SW620 and HCT116 cells, overexpression of circXRN2 upregulated viability of SW480 cells. (**G***–***I**) EdU assays of SW620, HCT116 and DLD-1 cells were performed to evaluate cell proliferation. (**J***–***L**) Flow cytometry of SW620, HCT116 and DLD-1 cells were performed to evaluate cell apoptosis. (**M***–***O**) Scratch assay of SW620, HCT116 and DLD-1 cells were performed to evaluate cell migration. (**P***–***R**) Transwell assay of SW620, HCT116 and DLD-1 cells were performed to evaluate cell migration and invasion. Experiments were performed in triplicate, and data were presented as means ± SD. The symbol “*” “**” denotes statistical significance compared to the control group (p < 0.05, p < 0.01), with n = 3 replicates.

To delve into the biological functions of circXRN2 in CRC cells, we synthesized short interfering RNAs (siRNAs) to suppress circXRN2 expression and constructed overexpression plasmids to augment circXRN2 levels (The sequences were shown in Table 3). The efficacy of circXRN2 depletion and overexpression was validated through qRT-PCR. As depicted in Fig. 2A–C, si-circXRN2-1 was employed to deplete circXRN2 in HCT116 cells, si-circXRN2-3 was utilized for knockdown in SW620 cells, and OE-circXRN2 was employed to achieve overexpression in DLD-1 cells.

**Table 3:** Primer Sequences of si-circXNR2-1, si-circXNR2-1, si-circXNR2-3 .

Name	Sense	Antisense
si-circXNR2-1	CAGAGGAUAAUGUCAGAAUAUTT	AUAUUCUGACAUUAUCCUCUGTT
si-circXNR2-2	GGAUAAUGUCAGAAUAUGUGGTT	CCACAUAUUCUGACAUUAUCCTT
si-circXNR2-3	GAGGAUAAUGUCAGAAUAUGUTT	ACAUAUUCUGACAUUAUCCUCTT
si-NC	UUCUCCGAACGUGUCACGUdTdT	ACGUGACACGUUCGGAGAAdTdT

We assessed the influence of circXRN2 on cell proliferation via CCK-8 and EdU assays (Fig. 2D–I). The results revealed that the downregulation of circXRN2 hindered proliferation, whereas overexpression of circXRN2 stimulated proliferation. To investigate the impact of circXRN2 on apoptosis in CRC cell lines, we conducted flow cytometry (Fig. 2J–L). These results indicated that circXRN2 downregulation promoted apoptosis, while overexpression of circXRN2 inhibited apoptosis.

Scratch assays were conducted to evaluate the effect of circXRN2 on cell migration (Fig. 2M–O). The findings demonstrated that circXRN2 downregulation impeded migration, whereas overexpression of circXRN2 facilitated migration. To further validate the influence of circXRN2 on cell migration and invasion, we performed Transwell migration and invasion assays (Fig. 2P–R). These assays revealed that circXRN2 downregulation inhibited both migration and invasion, while overexpression of circXRN2 augmented both processes. Collectively, these results established that circXRN2 played an oncogenic role in CRC cells.

### circ-XRN2 promotes proliferation and metastasis of CRC cells in vivo (xenograft and liver-lung metastasis models)

**Figure 3:**
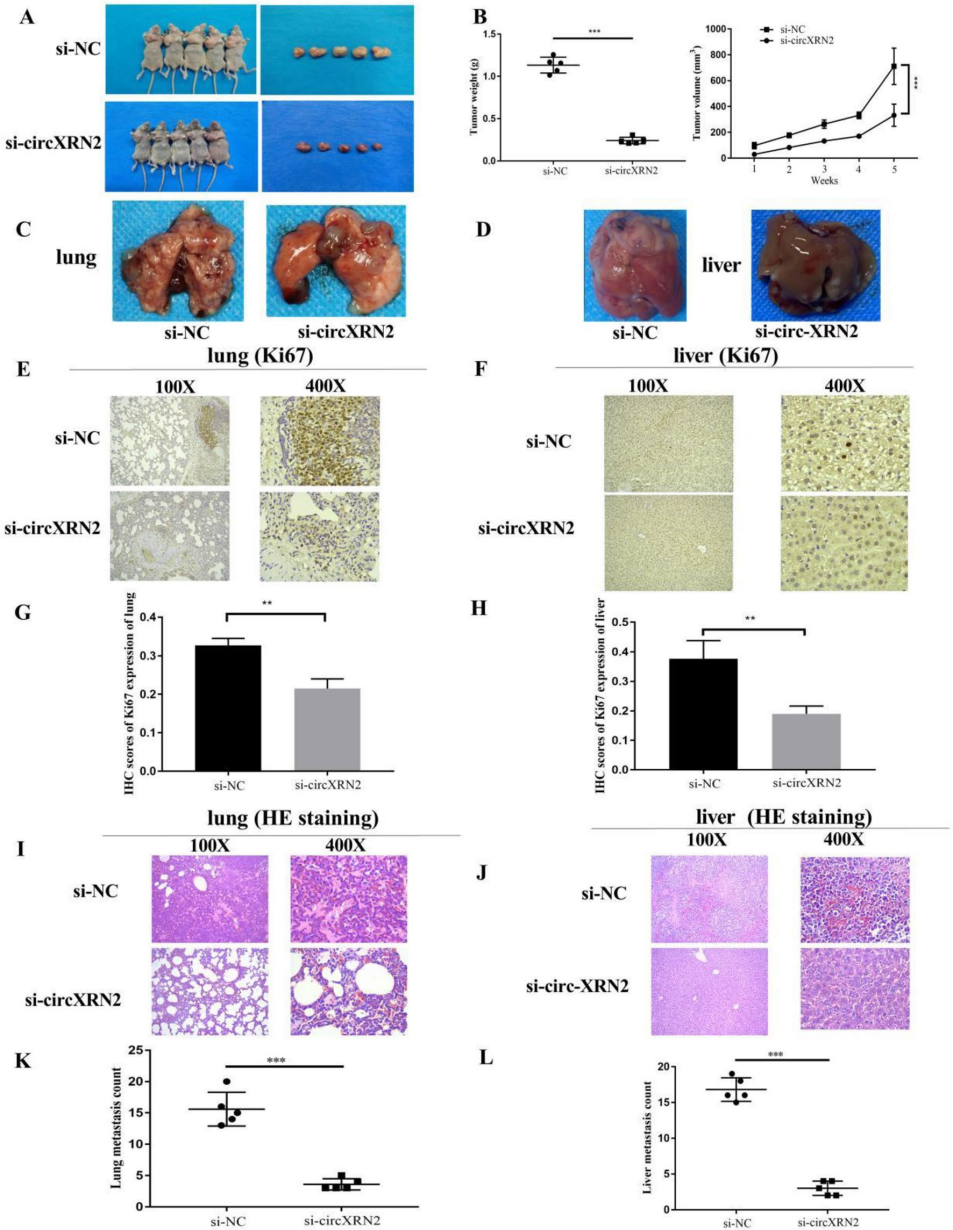
circ-XRN2 promotes proliferation and metastasis of CRC cells in vivo (xenograft and liver-lung metastasis models). (**A**) Representative picture of subcutaneous xenograft tumors (n = 5 for each group). (**B**) Curves of tumor weights and volumes show negative effects of circXRN2 knockdown on the formation of subcutaneous xenograft tumors. (**C**,**D**) Images of visible nodules on the lung and liver surface. (**E**–**H**) Images and statistics of Ki-67 IHC staining on the lung and liver surface. (**I**–**L**) Representative images and HE staining of metastatic nodules in the lungs and livers of mice. **p < 0.01, ***p < 0.001, vs the control group, n = 5.

To further substantiate the effects of circ-XRN2 on in vivo tumor growth, we established stable transfections of HCT116 cells with si-circXRN2 and the corresponding negative control (si-NC). These cells were subsequently subcutaneously injected into BALB/c nude mice, and we monitored tumor volume at 7-day intervals over a span of 35 days. In the xenograft model, the data unequivocally demonstrated that circXRN2 depletion exerted a significant inhibitory effect on tumor growth (Fig. 3A). This inhibition was reflected in reduced tumor weight and volume when compared to the control group (Fig. 3B).

For the tail vein tumor metastasis model, we closely examined the lungs and liver to evaluate the impact of circXRN2 depletion on metastasis (Fig. 3C,D). Immunohistochemistry results clearly revealed that Ki67 expression was significantly lower in the circXRN2 knockdown group compared to the control group (Fig. 3E–H). Furthermore, H&E staining disclosed a notable reduction in the number of metastatic nodules in both the lungs and liver of the circXRN2 knockdown group (Fig. 3I–L). Taken together, these findings offered compelling evidence that circXRN2 indeed promoted in vivo proliferation and metastasis of HCT116 cells.

### circXRN2 functions as a sponge for miR-149-5p

**Figure 4:**
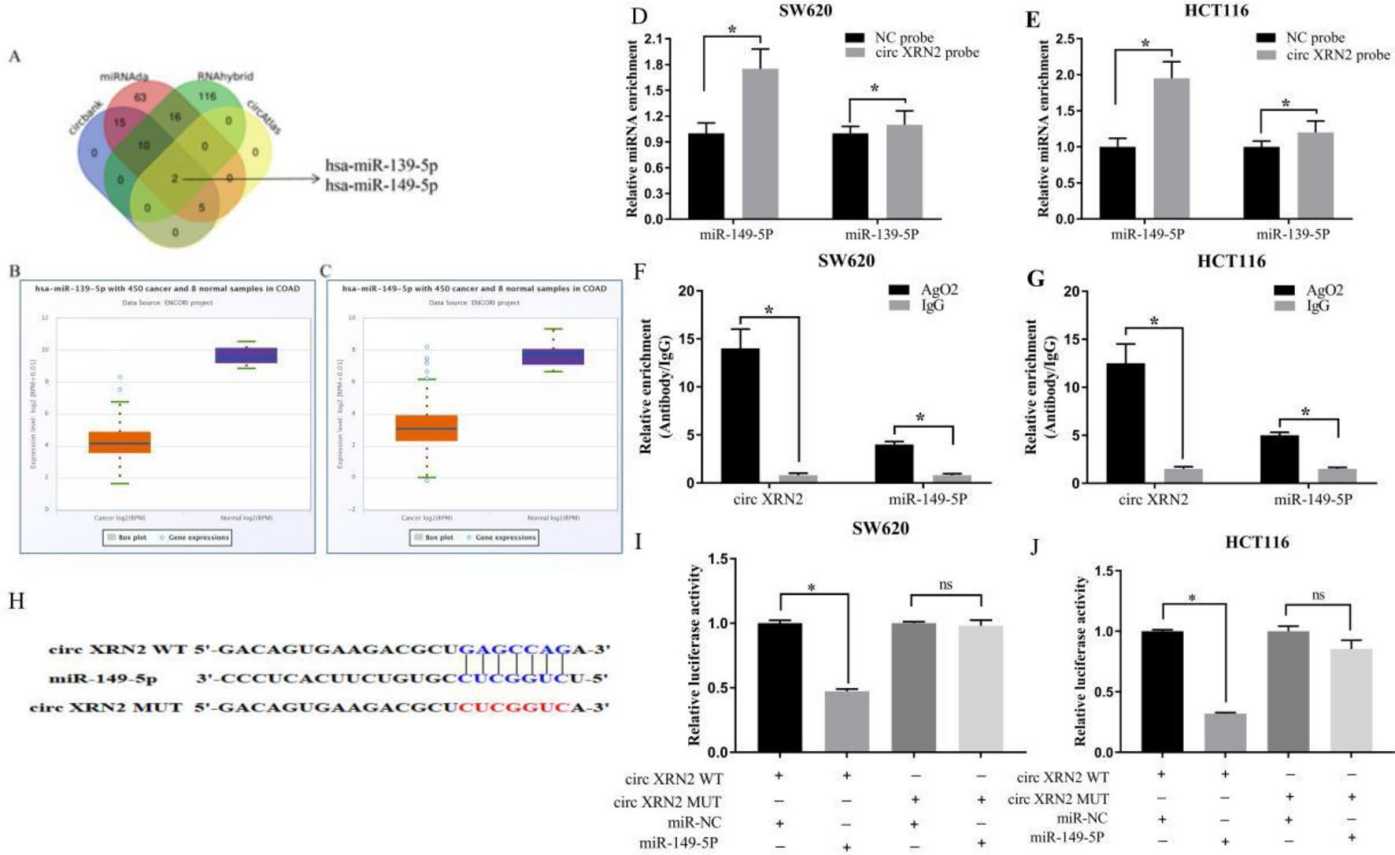
circXRN2 functions as a sponge for miR-149-5p. (**A**) Venn diagram showing the overlap of the target miRNAs of circXRN2 predicted by miRanda, circbank, RNAhybrid and circAtlas. (**B**,**C**) The targetscan database was used to analyze the expression of miR-139-5p and miR-149-5p in CRC tissues. (**D**,**E**) The expression levels of miR-139-5p and miR-149-5p were detected in the RNAs pulled down by circXRN2 and oligo probes. (**F**,**G**) Anti-AGO2 RIP was performed to detect circXRN2 and miR-149-5p in SW620 and HCT116 cells. (**H**) The online tool StarBase 2.0 was used to predict the binding sites between circXRN2 and miR-149-5p. (**I**,**J**) A luciferase reporter assay was used to confirm the interaction between circXRN2 and miR-149-5p in SW620 and HCT116 cells.

Previous research suggests that circXRN2 primarily localizes to the cytoplasm. Therefore, we hypothesized that circXRN2 might function as a sponge for miRNAs in CRC. To identify potential downstream miRNAs of circXRN2, we utilized four miRNA target prediction databases (circBank, miRNAda, RNAhybrid, and circAtlas) to screen for candidate miRNAs that could potentially bind to circXRN2. Two miRNAs, miR-139-5p and miR-149-5p, were selected (Fig. 4A–C). We employed biotin-labeled circXRN2 for RNA pull-down analysis to identify potential binding miRNAs, and miR-149-5p was found to be the most highly enriched miRNA in the sponge complex in both CRC cell lines (Fig. 4D,E).

It has been reported that miRNAs function as part of the RNA-induced silencing complex (RISC) by binding to Ago2. To validate the interaction between circXRN2 and miR-149-5p, we constructed an anti-Ago2 RIP assay. The results showed that both circXRN2 and miR-149-5p were significantly enriched in the anti-Ago2 antibody pull-down fraction (Fig. 4F,G). Additionally, dual-luciferase reporter gene assays were conducted in two CRC cell lines (SW620 and HCT116) to confirm the interaction between circXRN2 and miR-149-5p. The results revealed that the miR-449b-5p mimic significantly reduced the luciferase activity of wild-type circXRN2, while it had no significant effect on mutant XRN2 (Fig. 4H–J). In summary, these results confirmed that circXRN2 functioned as a sponge for miR-149-5p.

### The role of miR-149-5p in CRC

**Figure 5:**
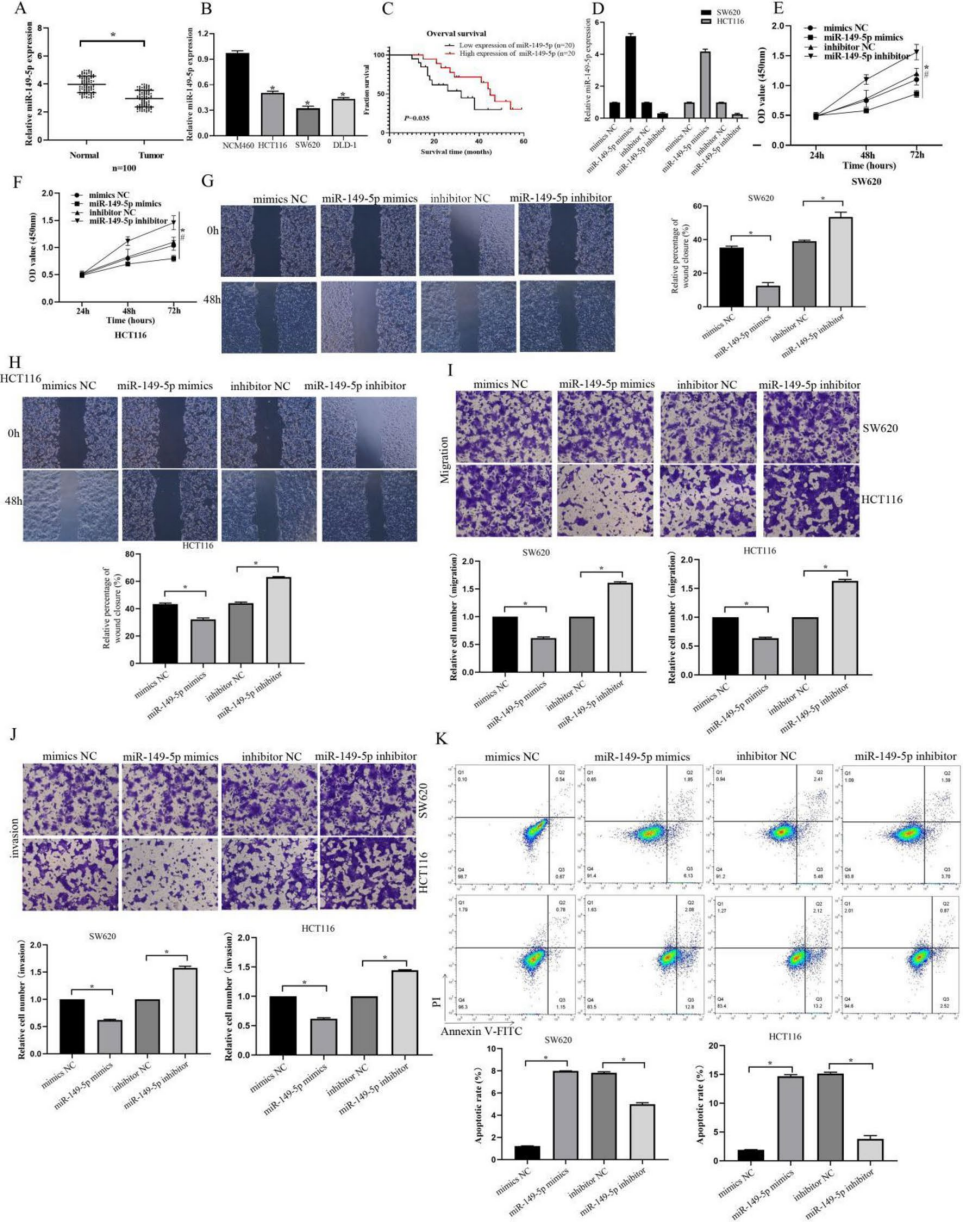
The role of miR-149-5p in CRC. (**A**,**B**) qRT-PCR analysis of miR-149-5p expression in tumor tissue and colorectal cancer cells. (**C**) Kaplan–Meier survival analysis with a Log rank test was performed to compare the overall survival rate of CRC patients with higher or lower level than median miR-149-5p expression level. (**D**) The construction of SW620 and HCT116 cells transfected with miR-149-5p mimics and miR-149-5p inhibitor. (**E**,**F**) CCK-8 assays of SW620 and HCT116 cells transfected with miR-149b-5p mimics and miR-149-5p inhibitor were performed to evaluate cell proliferation. (**G**,**H**) Wound healing assays of SW620 and HCT116 cells transfected with miR-149b-5p mimics and miR-149-5p inhibitor were performed to evaluate cell migration. (**I**,**J**) Transwell assays of SW620 and HCT116 cells transfected with miR-149b-5p mimics and miR-149-5p inhibitor were performed to evaluate cell migration and invasion. (**K**) Flow cytometry of SW620 and HCT116 cells transfected with miR-149b-5p mimics and miR-149-5p inhibitor were performed to evaluate cell apoptosis. *p < 0.05, vs the control group, n = 5.

Next, we explored the expression of miR-149-5p in tissues. Compared to adjacent normal tissues, miR-149-5p expression was significantly downregulated in CRC tissues (Fig. 5A). To determine the functional role of miR-149-5p, we examined the expression of miR-149-5p in human normal colonic epithelial cells (NCM460) and CRC cells (HCT116, DLD-1, and SW620). We found that miR-149-5p was significantly downregulated in CRC cells (Fig. 5B). Furthermore, patients with higher levels of miR-149-5p tended to have longer overall survival (Fig. 5C). Subsequently, we established miR-149-5p knockdown and overexpression cell lines (Fig. 5D). CCK-8 assay showed that overexpression of miR-149-5p significantly inhibited the proliferation of CRC cells, while depletion of miR-149-5p significantly promoted cell proliferation (Fig. 5E,F). Scratch migration assay demonstrated that overexpression of miR-149-5p significantly inhibited the migration of CRC cells, while depletion of miR-149-5p significantly promoted cell migration (Fig. 5G,H). Transwell migration and invasion assay indicated that overexpression of miR-149-5p significantly inhibited the migration and invasion of CRC cells, while depletion of miR-149-5p significantly promoted both migration and invasion (Fig. 5I,J). Flow cytometry apoptosis assay showed that overexpression of miR-149-5p significantly promoted apoptosis in CRC cells, while depletion of miR-149-5p significantly inhibited apoptosis (Fig. 5K).

### circXRN2 exerts pro-tumor effects in CRC by targeting miR-149-5p

**Figure 6:**
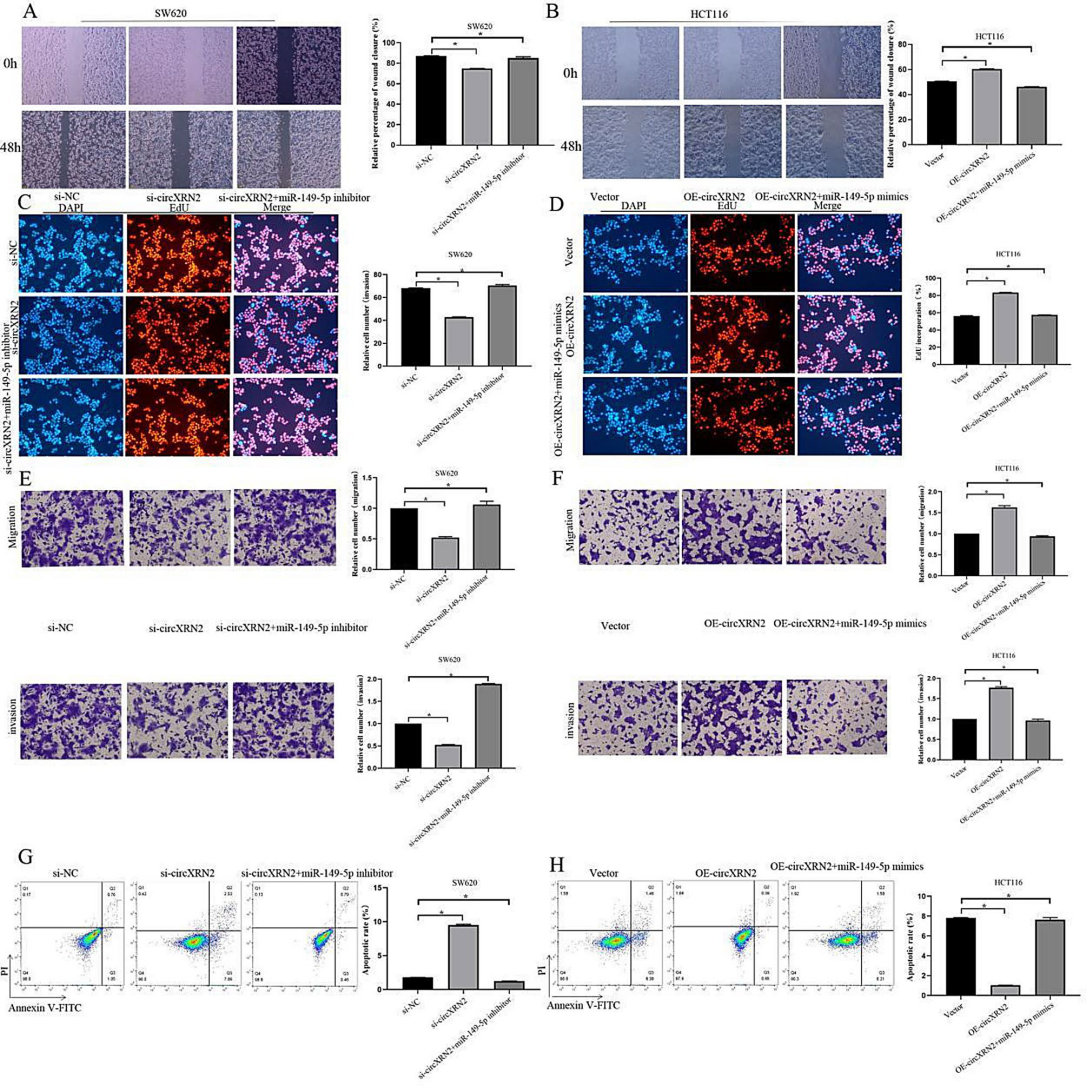
circXRN2 exerts antitumor effects in CRC by targeting miR-149-5p. Wound healing assays (**A**,**B**), EdU assays (**C**,**D**)**,** Transwell assays (**E**,**F**) and Flow cytometry (**G**,**H**) was conducted to assess the migration, proliferation, invasion and apoptosis of CRC cells transfected with si-NC, si-circXRN2, si-circXRN2 + miR-149-5p inhibitor and Vector, OE-circXRN2, OE-circXRN2 + miR-149-5p mimics. *p < 0.05, vs the control group, n = 5.

To further delve into the roles of miR-149-5p and circXRN2 in CRC progression, we conducted a series of rescue experiments. The results from the scratch migration assay revealed that the miR-149-5p inhibitor could reverse the inhibitory effect of circXRN2 depletion on SW620 cell migration (Fig. 6A), while the miR-149-5p mimic could counteract the promoting effect of circXRN2 overexpression on HCT116 cell migration (Fig. 6B). The EdU assay results further supported these findings, indicating that the miR-149-5p inhibitor could restore the suppressed proliferation of SW620 cells caused by circXRN2 downregulation (Fig. 6C), while the miR-149-5p mimic could counter the enhanced proliferation of HCT116 cells induced by circXRN2 overexpression (Fig. 6D). Additionally, the results from the Transwell migration and invasion assays showed that the inhibitory effects of circXRN2 knockdown on migration and invasion could be reversed by the miR-149-5p inhibitor (Fig. 6E), and transfection with the miR-149-5p mimic could counteract the promoting effect of circXRN2 overexpression (Fig. 6F).

We also examined apoptosis. Flow cytometry demonstrated that the miR-149-5p inhibitor could counteract the apoptosis-promoting effect of circXRN2 downregulation in SW620 cells (Fig. 6G), while the miR-149-5p mimic could reverse the apoptosis-inhibiting effect of circXRN2 overexpression in SW620 cells (Fig. 6H). In summary, these data strongly suggested that miR-149-5p played a pivotal role downstream of circXRN2 in the context of CRC progression.

### Identification of downstream target genes of miR-149-5p

**Figure 7:**
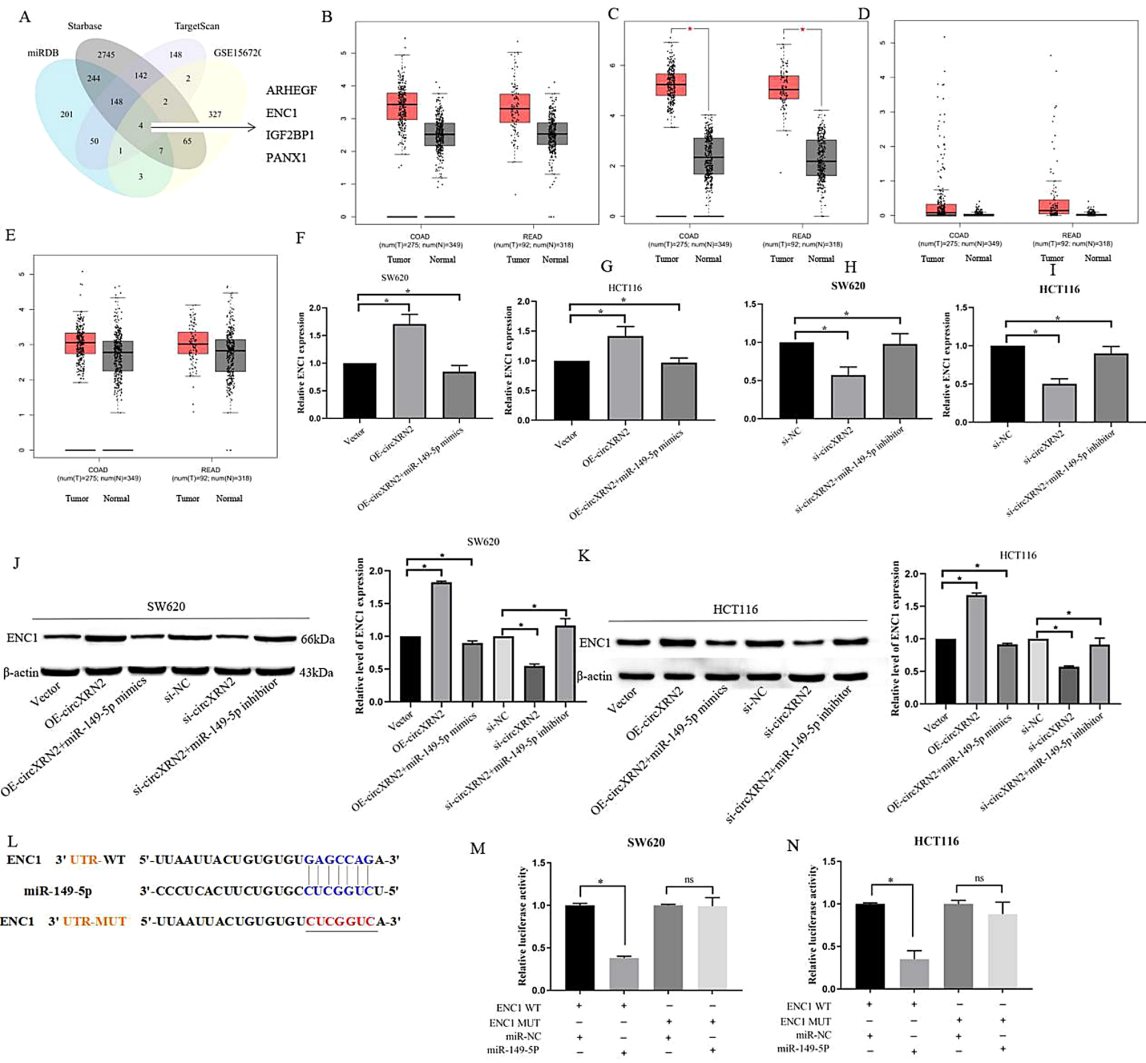
Identification of downstream target genes of miR-149-5p. (**A**) Target mRNAs of miR-149-5p was predicted by miRDB, Starbase, Targetscan and upregulated genes in CRC tissues compared to adjacent normal tissue based on the GEO dataset (GSE156720) were shown in Venn diagram. (**B**–**E**) The GEPIA database was used to analyze the expression of ARHEGF, ENC1, IGF2BP1 and PANX1 in CRC tissues (COAD stands for colon adenocarcinoma and READ for rectal adenocarcinoma). (**F**–**K**) The expression of ENC1 at the mRNA and protein levels was positively regulated by circXRN2. Importantly, this regulation could be fully restored by the introduction of either the miR-149-5p mimic or inhibitor. (**L**) The online tool StarBase 2.0 was employed to predict the binding sites of miR-149-5p in ENC1 3ʹUTR. (**M**,**N**) Dual-luciferase reporter assay was used to analyze the interaction between ENC1 and miR-149-5p in CRC cells.

To further elucidate the role of miR-149-5p in CRC cells, we employed three online databases (miRDB, StarBase, TargetScan) to predict potential target genes to which miR-149-5p could bind in the 3′UTR. By combining this information with the upregulated genes identified from the GSE156720 database, we identified four genes common to all four databases (ARHGEF39, ENC1, IGF2BP1, and PANX1) (Fig. 7A). We then utilized the GEPIA database (http://gepia.cancerpku.cn/detail.php) to further refine our search for genes that exhibited a negative correlation with miR-149-5p expression in CRC (Fig. 7B–E). Among these genes, only ENC1 demonstrated a significant increase in expression in CRC tissues compared to adjacent normal tissues, with statistical significance. Our investigation revealed that the expression of ENC1 at the mRNA and protein levels was positively regulated by circXRN2. Importantly, this regulation could be fully restored by the introduction of either the miR-149-5p mimic or inhibitor (Fig. 7F–K).

To validate the target relationship between miR-149-5p and ENC1, we conducted dual-luciferase reporter assays. As illustrated in Fig. 7L, we inserted the 3′UTR sequences of ENC1, containing either wild-type or mutant miR-149-5p binding sites, into the luciferase reporter vector. The results indicated that transfection with miR-149-5p led to a reduction in luciferase activity in the ENC1 3′UTR-WT group but had no significant effect on the MACC1 3′UTR-MUT group (Fig. 7M,N). In summary, these findings suggested that circXRN2 might exert its effects by upregulating ENC1 through miR-149-5p sponging.

### circXRN2 depletion suppresses CRC progression through the miR-149-5p/ENC1 axis

**Figure 8:**
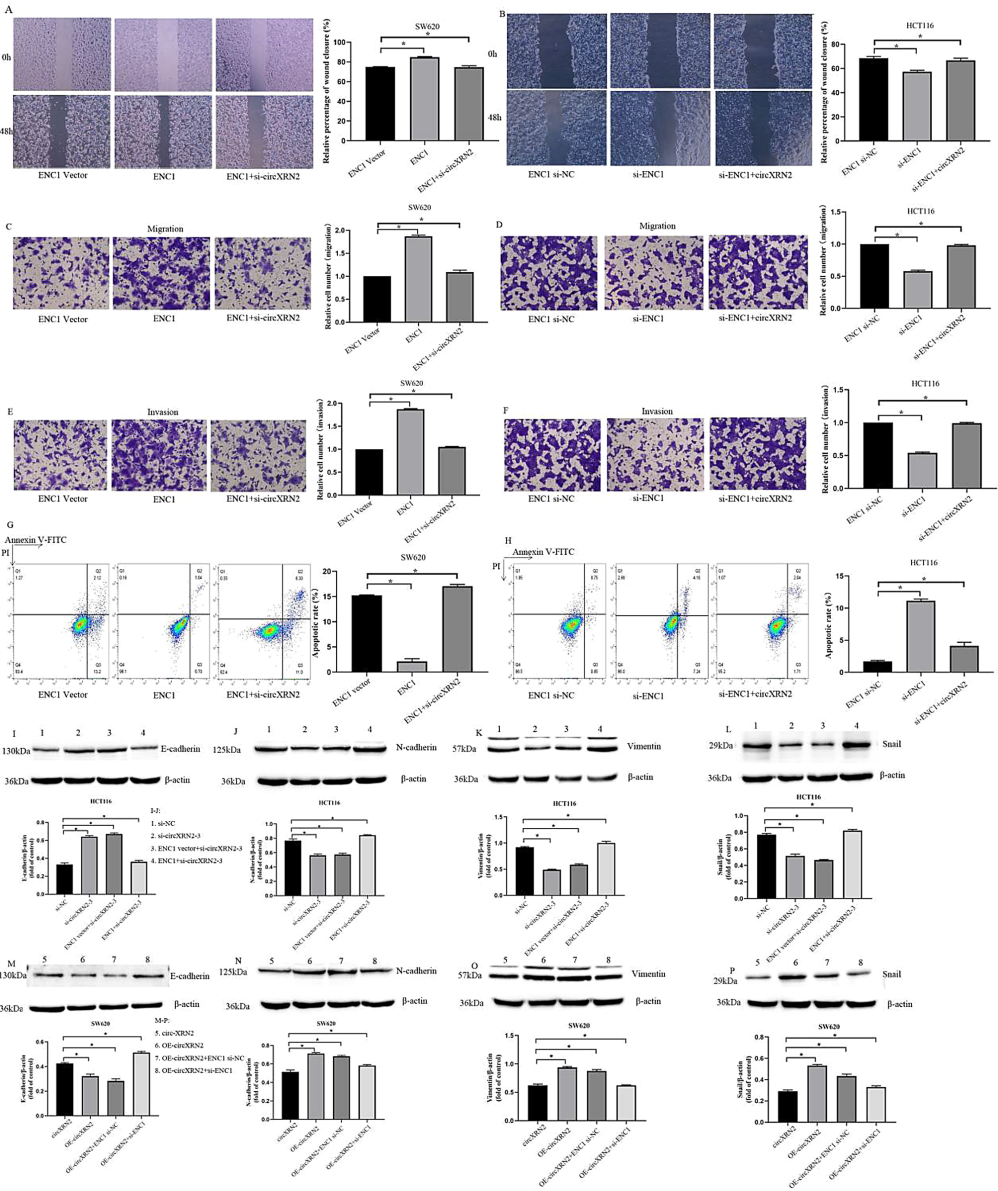
circXRN2 depletion suppresses CRC progression through the miR-149-5p/ENC1 axis. Wound healing assays (**A**,**B**), Transwell assays (**C**–**F**) and Flow cytometry (**G**,**H**) was conducted to assess the migration, invasion and apoptosis of CRC cells transfected with ENC1 vector, ENC1, ENC1 + si-circXRN2 and ENC1 si-NC, si-ENC1, si-ENC1 + circXRN2. *p < 0.05, vs the control group, n = 5. (**I**–**L**) Western blot results demonstrated that in HCT116 cells transfected with si-circXRN2, the protein levels of EMT markers, such as N-cadherin, Vimentin, and Snail, decreased, while the protein expression of the epithelial marker E-cadherin increased. Importantly, overexpression of ENC1 was able to reverse these changes. (**M**–**P**) In SW620 cells transfected with OE-circXRN2, the protein levels of EMT markers, such as N-cadherin, Vimentin, and Snail, increased, while the protein expression of the epithelial marker E-cadherin decreased. Once again, overexpression of ENC1 was able to counteract these effects. *p < 0.05, vs the control group, n = 3.

Results from both the scratch migration assays and Transwell migration and invasion assays revealed that the overexpression of ENC1 promoted the migration and invasion of CRC cells, while the depletion of ENC1 inhibited these processes. Notably, these effects could be reversed by either circXRN2 depletion or overexpression (Fig. 8A–F). Flow cytometry further demonstrated that the overexpression of ENC1 suppressed apoptosis in CRC cells, while depletion of ENC1 promoted apoptosis. Importantly, these effects on apoptosis could be counteracted by manipulating circXRN2 expression (Fig. 8G,H). In summary, we proposed that circXRN2 inhibited CRC progression by upregulating ENC1 expression through miR-149-5p sponging. Metastasis is a major contributor to cancer incidence and mortality, and the EMT is a process through which epithelial cells acquire a mesenchymal stem cell-like phenotype. Numerous studies have established the involvement of EMT in tumor cell metastasis^12,13^. To investigate the impact of circXRN2 on EMT-related proteins, we employed Western blotting analysis. The results demonstrated that in HCT116 cells transfected with si-circXRN2, the protein levels of EMT markers, such as N-cadherin, Vimentin, and Snail, decreased, while the protein expression of the epithelial marker E-cadherin increased. Importantly, overexpression of ENC1 was able to reverse these changes (Fig. 8I–L). Conversely, in SW620 cells transfected with OE-circXRN2, the protein levels of EMT markers, such as N-cadherin, Vimentin, and Snail, increased, while the protein expression of the epithelial marker E-cadherin decreased. Once again, overexpression of ENC1 was able to counteract these effects (Fig. 8M–P). Therefore, these findings suggested that circXRN2 could promote colorectal cancer progression via the miR‐149‐5p/ENC1 axis by inducing EMT process.

## Discussion

CRC presents a significant public health challenge due to its high incidence rate and often unfavorable prognosis. Understanding the mechanisms underlying its tumorigenesis and progression is crucial for improving diagnostic and therapeutic strategies. Similar to many other cancers, the precise pathogenesis of CRC remains incompletely understood. In recent years, mounting evidence has pointed to the pivotal role of circRNAs in initiating and driving cancer progression. CircRNAs have been found to exert regulatory control over various cellular functions, making them potential biomarkers and therapeutic targets in cancer^7,14^.

In our study, we conducted a comprehensive transcriptome analysis of CRC tissues and adjacent normal tissues from five pairs of CRC patients. Through this analysis, we identified circXRN2 as highly expressed in CRC tissues, and its elevated expression was associated with adverse prognoses in CRC patients. Subsequent in vitro experiments, including knockdown and overexpression assays, revealed that circXRN2 promoted the proliferation, migration, and invasion capabilities of CRC cells while inhibiting apoptosis. In vivo experiments, which included tumor xenograft and liver-lung metastasis models, further confirmed that depletion of circXRN2 restrained tumor size and volume, concurrently inhibiting metastasis to the liver and lungs. These collective findings underscored the oncogenic role of circXRN2 in CRC and highlighted its potential as a prognostic indicator for CRC patients.

The role of the circRNA-miRNA-target gene signaling axis in the development and progression of CRC has been elucidated. For instance, hsa_circ_0026416 has been shown to promote cell proliferation and migration in CRC by modulating miR-346 and the nuclear factor NFIB^15^. Additionally, hsa_circ_0106714 inhibits the proliferation, migration, and invasion of CRC cells by regulating the miR-942-3p/DLG2 axis^16^.

In our study, we employed four miRNA target prediction databases (circBank, miRNAda, RNAhybrid, and circAtlas) to screen for two candidate miRNAs, miR-139-5p and miR-149-5p, which might interact with circXRN2. Through RNA pull-down experiments, RIP assays, and dual-luciferase reporter assays, we ultimately confirmed the binding interaction between circXRN2 and miR-149-5p.

miRNAs, which are small non-coding RNA molecules approximately 22 nucleotides long, play pivotal regulatory roles in cancer diagnosis, development, and treatment^15,16^. In our present study, we observed a downregulation of miR-149-5p expression in both CRC tissues and CRC cells, and this downregulation was associated with poor patient prognosis. Overexpression of miR-149-5p significantly inhibited the proliferation, migration, and invasion of CRC cells, while the depletion of miR-149-5p had the opposite effect. Moreover, miR-149-5p overexpression promoted apoptosis in CRC cells, whereas miR-149-5p depletion suppressed apoptosis. In rescue experiments, we demonstrated that a miR-149-5p inhibitor could reverse the inhibitory effects of circXRN2 depletion on SW620 cell migration, proliferation, and invasion, while a miR-149-5p mimic could reverse the promotive effects of circXRN2 overexpression on HCT116 cell proliferation, migration, and invasion. These rescue experiments also confirmed that miR-149-5p could reverse the pro-apoptotic effects of circXRN2 on CRC cells.

By utilizing the miRDB, StarBase, and TargetScan databases, we screened for downstream targets of miR-149-5p. Combining this information with upregulated genes from the GSE156720 database, we identified ENC1 as a gene that was negatively correlated with miR-149-5p expression. Our findings revealed that circXRN2 positively regulated both the mRNA and protein levels of ENC1, and this regulation could be fully rescued by miR-149-5p mimics or inhibitors. Dual-luciferase reporter assays further confirmed the binding interaction between miR-149-5p and ENC1. Overexpression of ENC1 promoted the migration and invasion of CRC cells, while its depletion suppressed these processes. Importantly, these effects could be rescued by manipulating circXRN2 expression. In summary, we proposed that circXRN2, by sequestering miR-149-5p and upregulating ENC1 expression, played a suppressive role in CRC progression.

Metastasis remains the primary driver of elevated cancer incidence and mortality. The process of EMT is a complex biological phenomenon that has been extensively implicated in cancer initiation and metastasis^17^. A hallmark of EMT is the reduction in E-cadherin levels and an increase in Vimentin expression^18^. In our present study, when we transfected si-circXRN2 into HCT116 cells, we observed a decrease in the protein levels of mesenchymal markers such as N-cadherin, Vimentin, and Snail, while the expression of the epithelial marker E-cadherin increased. Importantly, overexpression of ENC1 was able to reverse these outcomes. Conversely, when we transfected OE-circXRN2 into HCT116 cells, we observed an increase in the protein expression levels of mesenchymal markers, including N-cadherin, Vimentin, and Snail, while the expression of the epithelial marker E-cadherin decreased. Once again, overexpression of ENC1 was able to counteract these effects. Therefore, our findings suggested that circXRN2 could activate the EMT signaling pathway in CRC progression through its modulation of ENC1.

## Conclusion

The results demonstrated that circXRN2 promoted the proliferation and metastasis of CRC cells through the miR-149-5p/ENC1/EMT axis, suggesting that circXRN2 might serve as a potential therapeutic target and novel biomarker in the progression of CRC.

### Supplementary Information

Supplementary Figures.

## Data Availability

The datasets generated and/or analysed during the current study are available from the corresponding author if necessary.
