# Supplementary material for: CircXRN2 accelerates colorectal cancer progression through regulating miR-149-5p/MACC1 axis and EMT
Source: Sci Rep. 2024 Jan 30;14:2448. doi: 10.1038/s41598-024-52257-3 (PMC10828403; doi:10.1038/s41598-024-52257-3)
Supplement: Supplementary file 1 — Supplementary Figures. [file 41598_2024_52257_MOESM1_ESM.pdf]

Fig1

F

SW620 **HCT116**

500 bp

200 bp

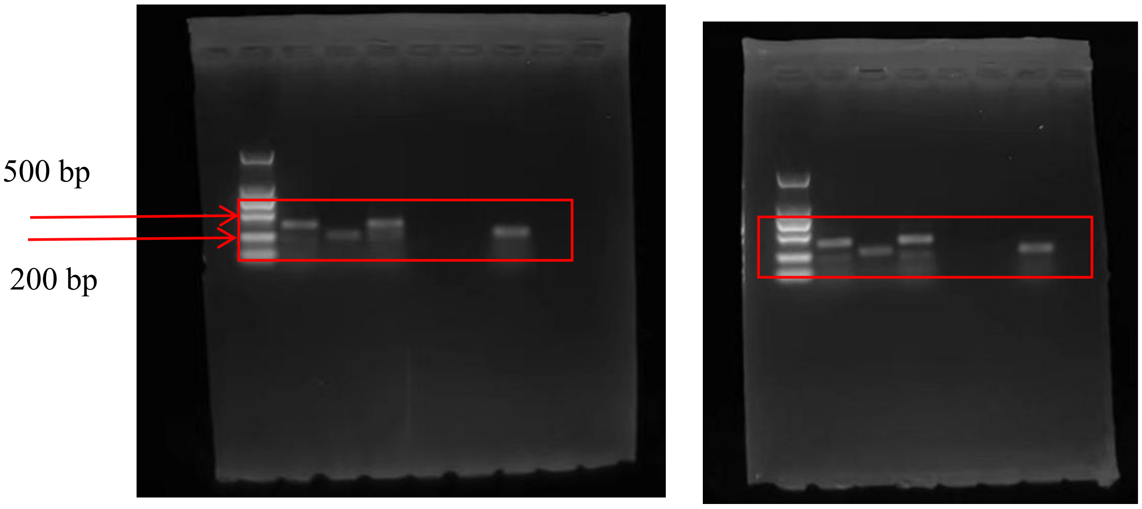

Fig7

J

SW620 ENC1

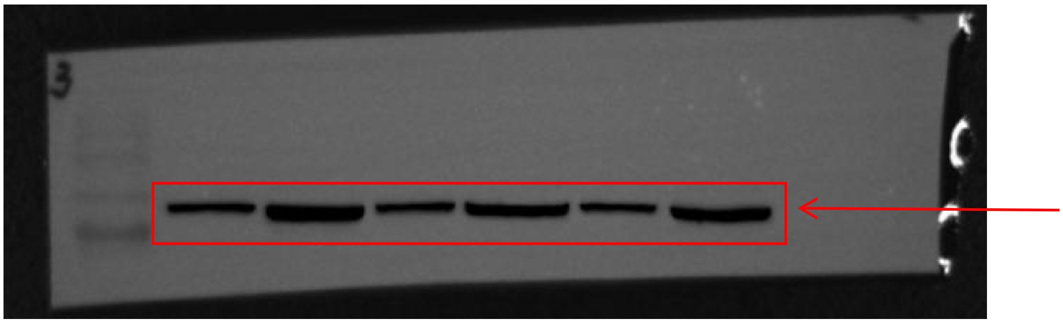

ENC1 66 kDa

SW620 β-actin

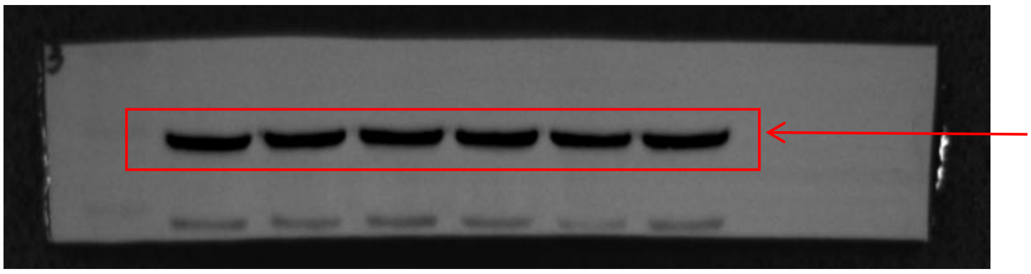

β-actin 43 kDa

K **HCT116 ENC1**

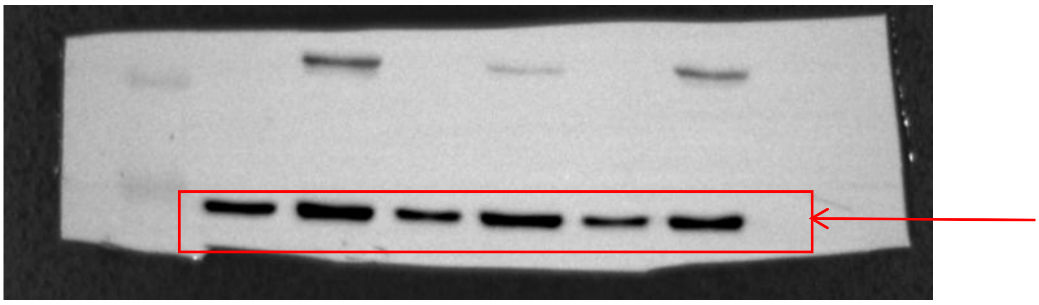

ENC1 66 kDa

HCT116 ENC1

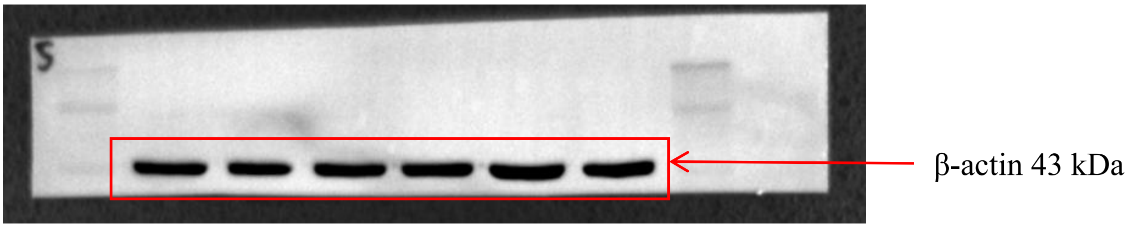

Fig8

I

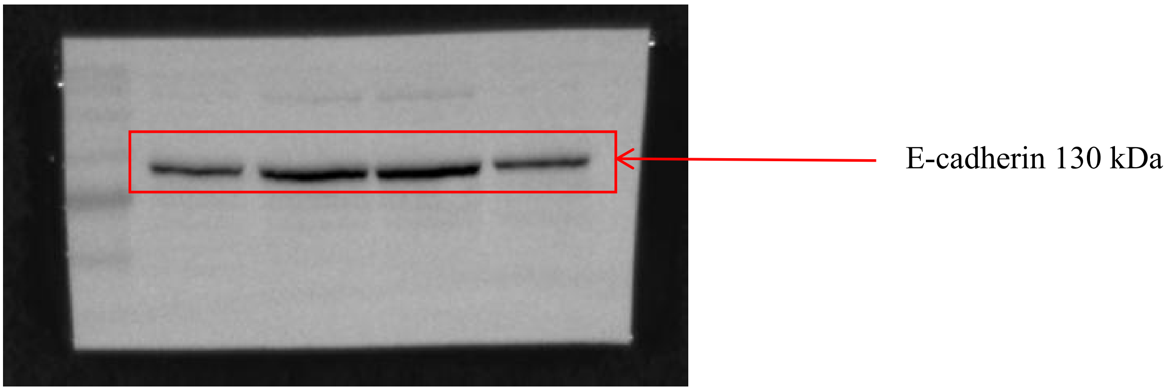

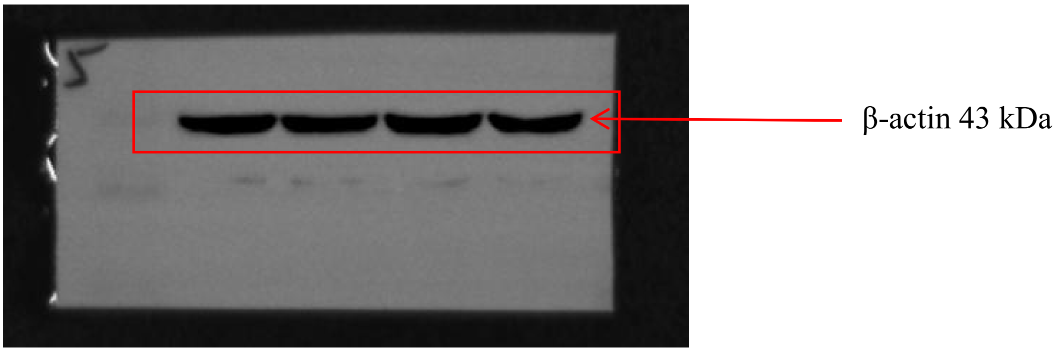

J

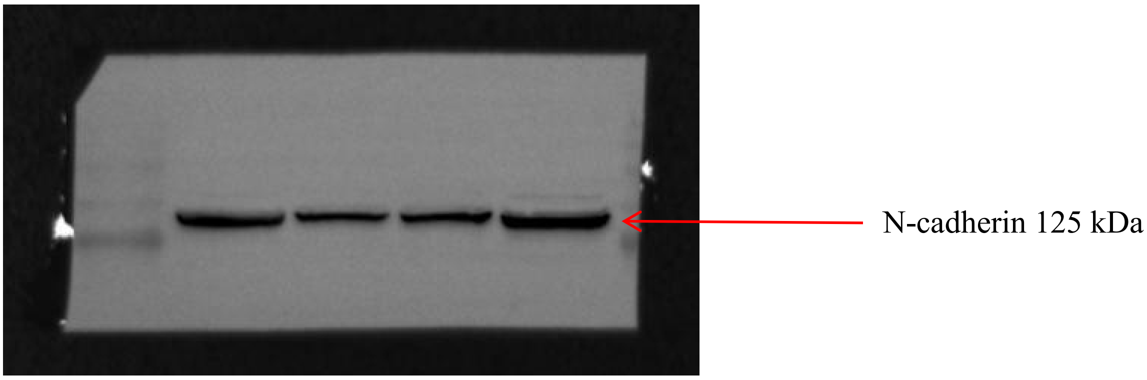

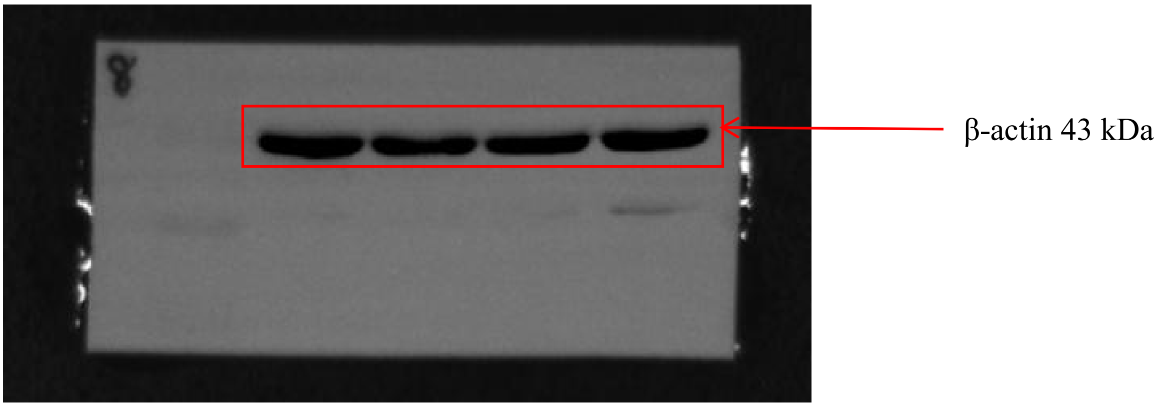

K

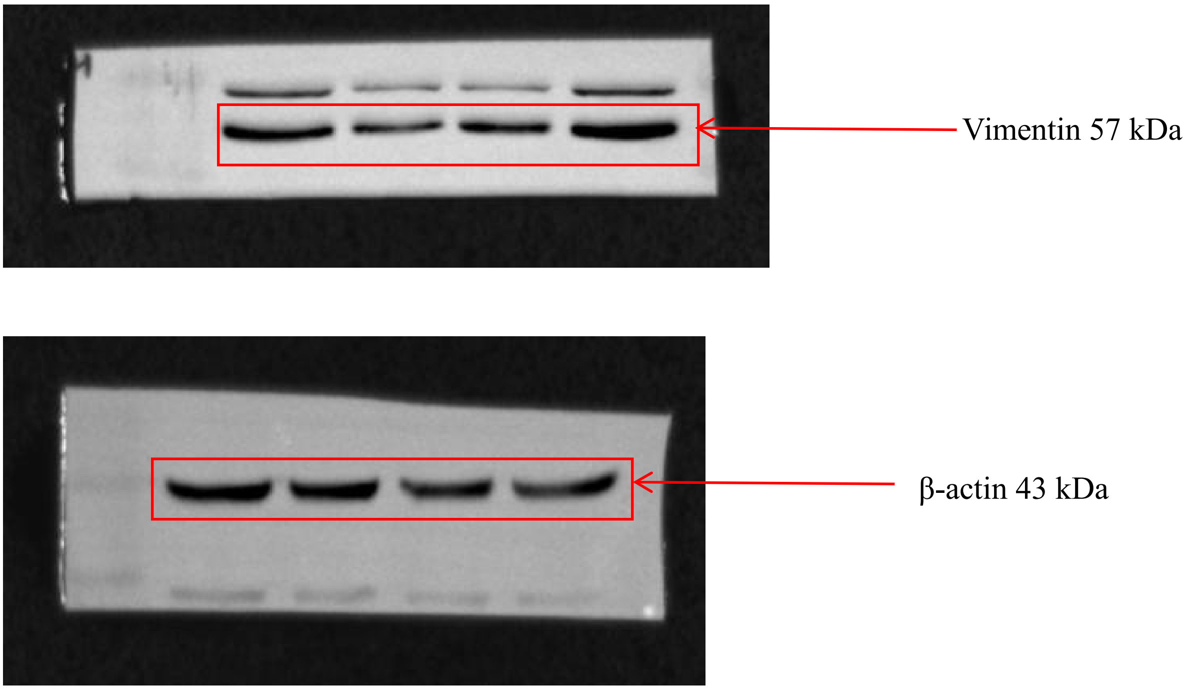

L

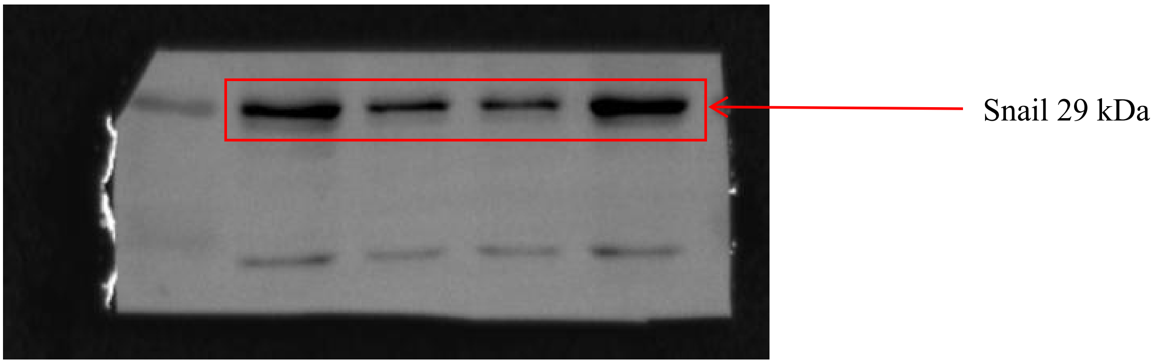

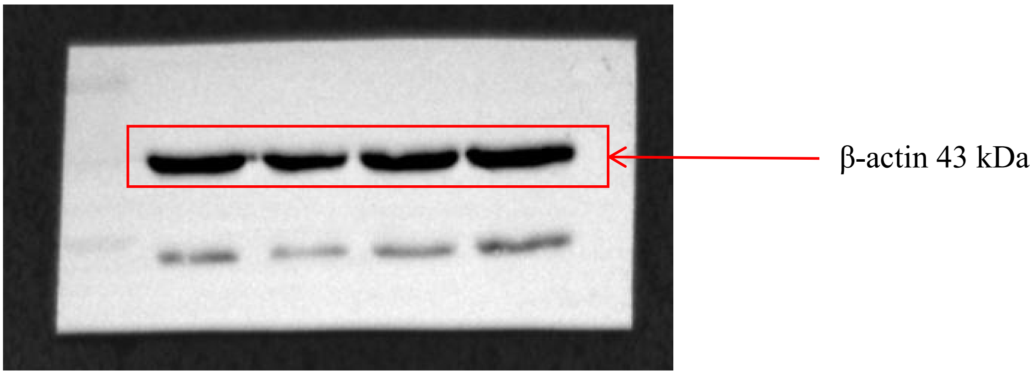
β-actin 43 kDa

M

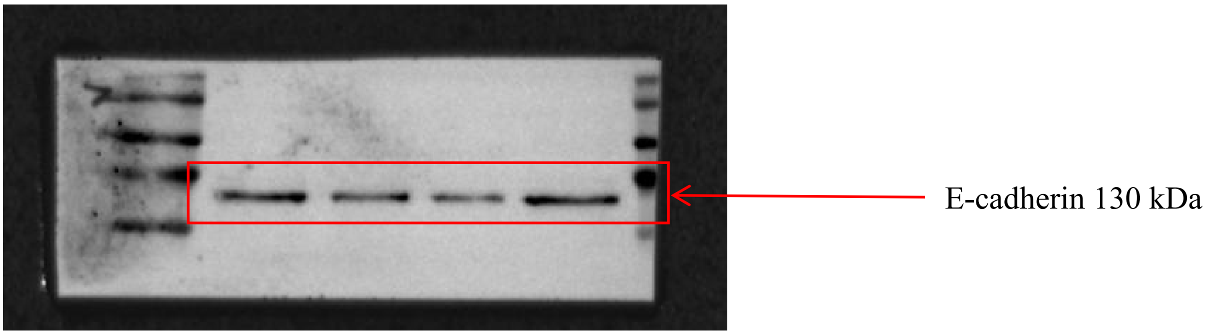
E-cadherin 130 kDa

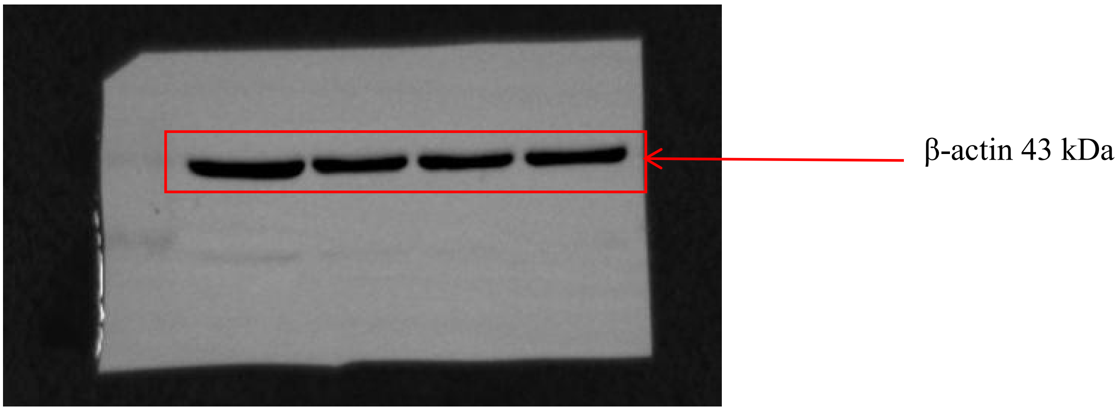
β-actin 43 kDa

N

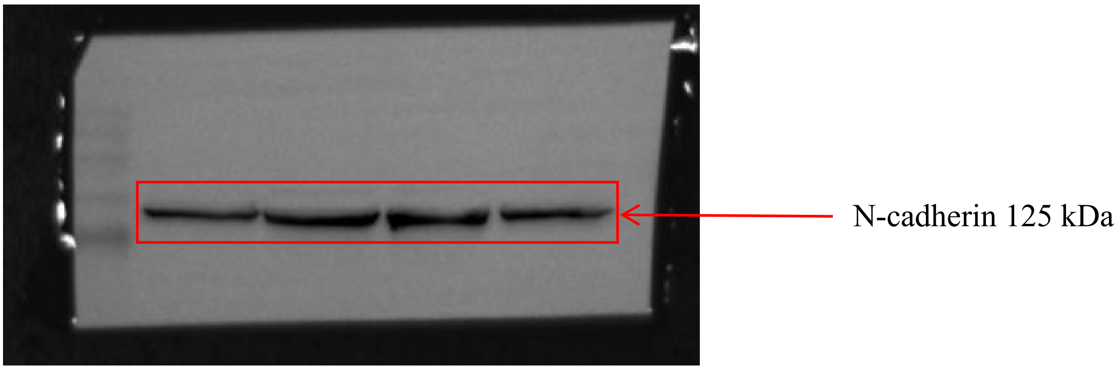
N-cadherin 125 kDa

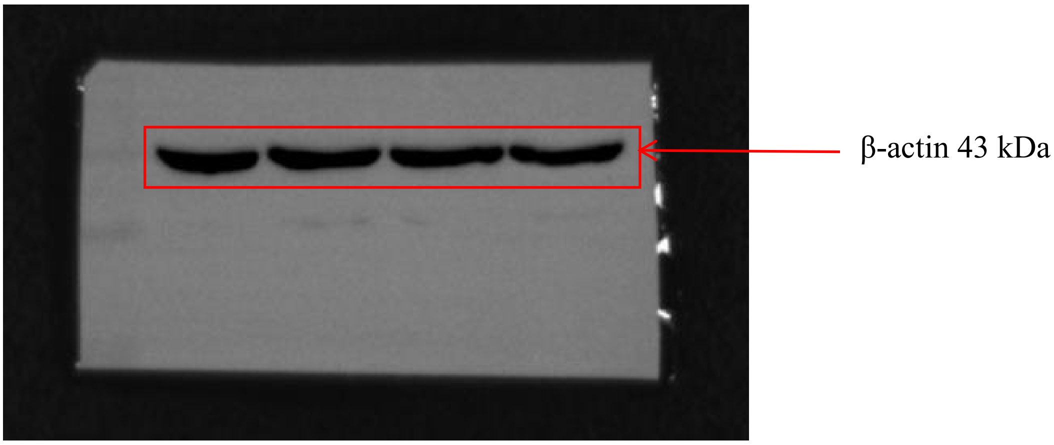

O

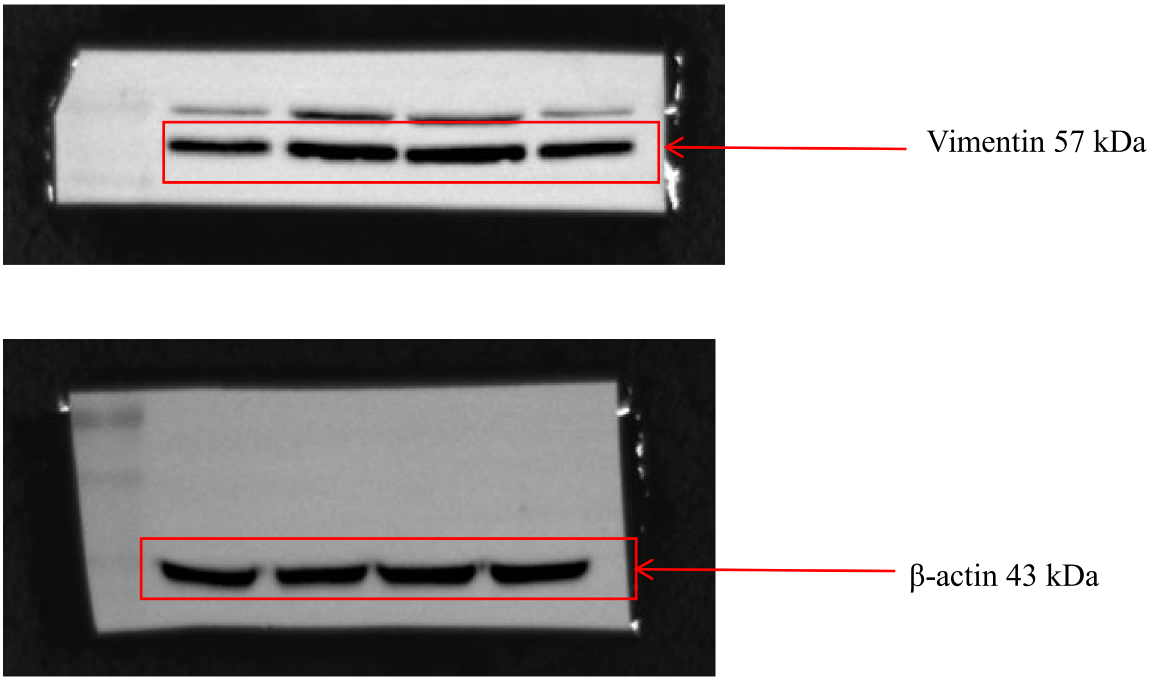

P

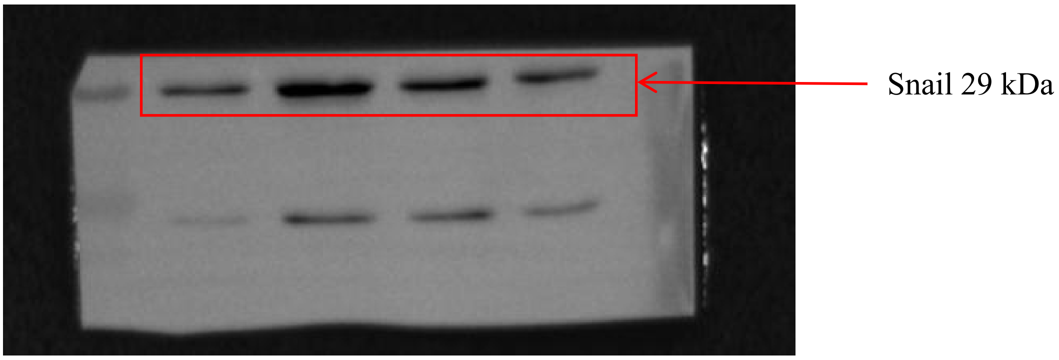

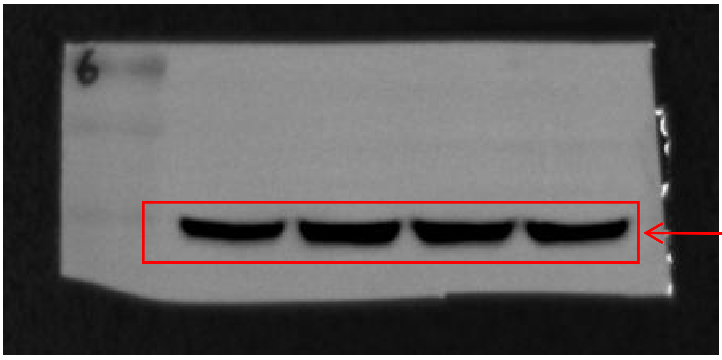

β-actin 43 kDa
